# The novel mechanism facilitating chronic hepatitis B infection: immunometabolism and epigenetic modification reprogramming

**DOI:** 10.3389/fimmu.2024.1349867

**Published:** 2024-01-15

**Authors:** Zhengmin Wang, Nan Liu, Yang Yang, Zhengkun Tu

**Affiliations:** ^1^ Department of Hepatology, The First Hospital of Jilin University, Changchun, Jilin, China; ^2^ Institute of Epigenetic Medicine, First Hospital of Jilin University, Changchun, China; ^3^ Institute of Liver Diseases, The First Hospital of Jilin University, Changchun, Jilin, China

**Keywords:** chronic hepatitis B infection, immunometabolism reprogramming, epigenetic modification, antiviral immunity, hepatic immune microenvironment

## Abstract

Hepatitis B Virus (HBV) infections pose a global public health challenge. Despite extensive research on this disease, the intricate mechanisms underlying persistent HBV infection require further in-depth elucidation. Recent studies have revealed the pivotal roles of immunometabolism and epigenetic reprogramming in chronic HBV infection. Immunometabolism have identified as the process, which link cell metabolic status with innate immunity functions in response to HBV infection, ultimately contributing to the immune system’s inability to resolve Chronic Hepatitis B (CHB). Within hepatocytes, HBV replication leads to a stable viral covalently closed circular DNA (cccDNA) minichromosome located in the nucleus, and epigenetic modifications in cccDNA enable persistence of infection. Additionally, the accumulation or depletion of metabolites not only directly affects the function and homeostasis of immune cells but also serves as a substrate for regulating epigenetic modifications, subsequently influencing the expression of antiviral immune genes and facilitating the occurrence of sustained HBV infection. The interaction between immunometabolism and epigenetic modifications has led to a new research field, known as metabolic epigenomics, which may form a mutually reinforcing relationship with CHB. Herein, we review the recent studies on immunometabolism and epigenetic reprogramming in CHB infection and discuss the potential mechanisms of persistent HBV infection. A deeper understanding of these mechanisms will offer novel insights and targets for intervention strategies against chronic HBV infection, thereby providing new hope for the treatment of related diseases.

## 1 Introduction

### 1.1 Hepatitis B virus and CHB infection

Chronic hepatitis B (CHB) infection poses a significant global health challenge affecting the health and well-being of millions of people (1). Hepatitis B Virus (HBV) is a partially double-stranded DNA virus with a sophisticated life cycle that can persist by forming covalently closed circular DNA (cccDNA) within the host cell nucleus. The structure of HBV comprises a lipid envelope, with its surface characterized by three types of surface proteins, namely small (S), medium (M), and large (L), collectively referred to as HBsAg in serology. Additionally, there is an internal core-shell enveloping the core particles, known as the hepatitis B core antigen (HBcAg) in serology. The outer membrane forms an icosahedral shell composed of 120 core protein dimers (2–5). cccDNA plays a crucial role in the subgenomic RNA mediated by host RNA polymerase II, encompassing the surface protein HBx and two RNA templates exceeding the genomic length, namely pregenomic (pg) RNA and precore RNA. These two types of RNA exert significant regulatory functions, leading to the generation of the precore protein precursor, ultimately resulting in the secretion of hepatitis B e antigen (HBeAg) (6). Sustained HBV infection has the potential to induce diverse levels of liver impairment, culminating in progression to chronic hepatitis, fibrosis, cirrhosis, and hepatocellular carcinoma (HCC) (7, 8). According to the World Health Organization (WHO), approximately 300 million individuals worldwide are afflicted with chronic HBV infection, and nearly one million succumb annually to complications directly linked to HBV infection (9).

The risk of developing CHB infection depends on the age at exposure, with infants and children being mostly symptom-free. Approximately 95% of newborns, 20–30% of children (1-5 years old), and < 5% of adults fail to clear the virus after six months (10). During CHB infection, the virus remains in the body for a long period. This can lead to severe liver damage, such as cirrhosis and liver cancer, with significant repercussions for affected individuals, their families, and society at large (11–13). Although antiviral drugs can reduce the viral load in patients with hepatitis B, a complete cure remains difficult to achieve (6). Therefore, scientists are attempting to understand the mechanisms underlying this disease more thoroughly and promptly.

HBV infection can cause liver damage and chronic diseases; however, the virus is not cytotoxic. The immune system plays a key role in controlling viral replication and liver injury (14–16). Patients who achieve HBeAg seroconversion spontaneously or after antiviral therapy show improved immune function (17, 18). This implies that combining immunotherapy with antiviral treatment may increase the likelihood of viral elimination (19–22). The immune response to HBV infection is complex and variable. Some studies have reported that immune activation leads to reduced HBV DNA levels and HBeAg seroconversion, indicating effective clearance of infected hepatocytes (23). Other studies have reported that the reduction in HBV DNA levels is only temporary, suggesting ineffective clearance (24). Therefore, the host immune response is a critical factor in determining the outcome of HBV infection (25). The mechanisms by which HBV modulates the hepatic immune microenvironment to promote chronic infection are not fully understood.

### 1.2 Hepatic immune microenvironment

Within the liver, there is a complex interplay among parenchymal (primarily hepatocytes), non-parenchymal, and recruited immune cells, collectively forming the hepatic immune microenvironment, which includes cellular components, secreted cytokines, and extracellular matrix components (26). One of the most intriguing features of the liver as a remarkable immune organ is its propensity to induce tolerance rather than immunity upon encountering foreign antigens (27). Specialized antigen-presenting cells in the liver, such as Kupffer cells and liver sinusoidal endothelial cells, are characterized by well-induced tolerance owing to their insufficient co-stimulatory signal transmission and tendency to produce immunosuppressive molecules, resulting in the establishment of an inherent hepatic tolerogenic microenvironment in a steady state (28). The nature of immune responses within the liver is intricately shaped by the level of inflammation. In cases of chronic or low-grade inflammation, where an immunosuppressive microenvironment prevails, the liver can serve as a “graveyard” for effector cells (29) or as a “school” for educating regulatory cells (30). These processes can lead to clonal deletion (31, 32) or suppression of antigen-specific T cells (33–35), which constitute the primary mechanisms underlying liver-induced antigen-specific tolerance. During CHB infection, HBV replication within hepatocytes goes unnoticed by the intracellular innate immune system, resulting in a tolerogenic hepatic microenvironment. This gradually leads to the alteration and deletion of the functional capacities of HBV-specific B and T cells, enabling the establishment of intractable persistent (chronic) HBV infection (36). However, how these mechanisms coordinate to sustain the tolerance of the hepatic microenvironment while the virus persists in the liver remains largely unknown (37). According to recent research in the relevant field, immunometabolism and epigenetic reprogramming have emerged as pivotal factors linking hepatocytes and immune cells within the hepatic immune microenvironment, as elaborated in the subsequent sections.

### 1.3 Immunometabolism and epigenetic modification

The immune system is intricately linked to metabolic functions in a manner that has not been previously recognized. This unconventional mode of thinking has been described in a novel field known as immunometabolism (38, 39). Immunometabolism encompasses a series of processes involving the energy metabolism of immune cells in response to pathogens, accumulation of metabolic products, and regulatory activity of metabolic enzymes (40). Reports of cellular metabolism being hijacked by viruses have a history spanning over half a century; however, the mechanisms and consequences of virus-induced metabolic reprogramming have only begun to be extensively studied over the past decade (41). Following internalization by glucose transporters, glucose molecules enter distinct metabolic pathways, including glycolysis and the pentose phosphate pathway (42, 43), each of which serves specific functions and purposes. To some extent, these changes influence the functionality of immune cells, thereby affecting the effectiveness of antiviral immune responses.

In addition to immunometabolism, epigenetic modifications play crucial roles in regulating gene expression (44, 45). Simultaneously, there is a vital interplay between metabolism and the epigenetic modification in immune regulation that disrupts the functionality and response patterns of immune cells (46, 47). The interaction between metabolism and epigenetics led to a new research field known as metabolic epigenomics (48). Recently emphasized metabolites such as lactate (49, 50), and a newly discovered epigenetic modification, lactylation (51), serve as noteworthy representatives. This combination also includes acetyl-CoA and histone acetylation (52, 53), S-adenosylmethionine and histone methylation (54, 55), as well as NAD^+^ and the Sirtuin family (56). These intricate interactive relationships play a pivotal role in the dynamic balance between immune responses and sustained viral infections.

During CHB infection, significant alterations occur in host immune metabolism, including glucose and lipid metabolism (57, 58). For instance, during HBV infection, cells involved in pathological responses, such as immune cells and hepatocytes, enhance utilization and glucose uptake to meet their energy and biosynthetic demands (50, 59). Concurrently, during CHB infection, epigenetic regulatory mechanisms such as DNA methylation and histone modification also undergo changes, subsequently influencing the expression patterns of specific genes (60, 61). Here, our aim is to systematically review the current state of research on immunometabolism and epigenetic modifications in CHB infection, as well as to anticipate their potential applications in future clinical treatments. By delving into these aspects of research, we hope to offer new perspectives and strategies for the treatment and prevention of HBV infection, making a positive contribution to the well-being of individuals affected by HBV infection worldwide.

## 2 Immunometabolism and epigenetic modification of HBV-infected hepatocytes

HBV affects various metabolic pathways within the liver to the extent that it is referred to as a metabolic virus (62). The liver assumes a pivotal function in the daily immune surveillance of the body (63). Its unique blood supply mechanism leads to continuous exposure to a variety of pathogens originating from both the bloodstream and gastrointestinal tract (64). Hepatocytes constitute the vast majority of liver masses (approximately 4/5) and play a role in various biochemical and metabolic functions (65). HBV infection leads to significant alterations in the host hepatocyte metabolic pathways and epigenetic modifications. These changes not only affect the bioenergetic supply of hepatocytes but also impact the immune response and viral replication in liver cells(as discussed subsequently).

### 2.1 Immunometabolism and antiviral immunity

The regulation of hepatocyte metabolism in CHB is an emerging and significant research area in the development of this disease. Hepatocytes not only act as active drivers of liver inflammation and fibrosis through intercellular communication but also engage in antiviral immunity by secreting type I interferons (IFNs) (66).

IFN serves as a critical initial defense mechanism that exerts control over viral gene replication and expression (67), with its expression directly influencing viral replication. Hepatocytes can trigger innate immune responses through pattern recognition receptors such as Retinoic Acid-Inducible Gene I (RIG-I)-like receptors (RLRs) (68, 69), which recruit the mitochondrial antiviral signaling (MAVS) adaptor (also known as VISA, Cardif, and IPS-1) (70, 71). Upon activation, MAVS molecules aggregate into virus-like supramolecular structures in the mitochondria (72, 73), initiating the activation of transcription factors including IRF3. This activation results in the production of IFN to execute antiviral activities (74, 75).

Recent pivotal studies have elucidated a novel mechanism by which HBV influences immune escape by regulating the immune metabolism of liver cells, thereby affecting their antiviral immune functions (57, 58, 76).

#### 2.1.1 Hepatocellular glycolysis

HBV can enhance the activity of the crucial glycolytic enzyme hexokinase 2 (HK2) in liver cells, promoting glycolysis. Furthermore, it stimulates lactate dehydrogenase A (LDHA), leading to increased lactate production; thus, facilitating the transition toward aerobic glycolysis (58), also known as the Warburg effect (77).

During this metabolic transition, HBV modulates the interplay between RIG-I and MAVS by orchestrating the activities of HK2 and lactate in a concerted manner. On one hand, HBV facilitates the assembly of a trimeric complex comprising HK2, MAVS, and voltage-dependent anion channel protein 1 (VDAC1) within the mitochondria, isolating MAVS from RIG-I and impeding their functional interaction. On the other hand, HBV elevates lactate expression, reinforcing its physical association with MAVS. This, in turn, impedes the interaction between RIG-I and MAVS, MAVS aggregation, and the subsequent signal transduction cascade, ultimately suppressing the production of INFs (58). Similarly, another recent study on metabolic immunity discovered that HBV can interact with the metabolic switch pyruvate kinase M2 isoform (PKM2) through the utilization of HBsAg, especially the large viral surface antigen (LHBS). This interaction inhibits the oligomerization of PKM2 within liver cells, leading to increased glucose consumption and lactate production, thereby promoting viral replication (57). The direct relationship between metabolic shifts and immune function can be explained by the lactate-MAVS-RIGI-IFN theory.

In summary, HBV regulates lactate production within liver cells through two mechanisms, thereby influencing antiviral immunity and promoting CHB infection. On one hand, HBV induces the formation of the HK2-MAVS-VDAC1 trimeric complex and promotes the interaction between lactate, a product of glycolysis, and MAVS. This impedes the interaction between MAVS and RIG-I, suppressing downstream signal transduction and ultimately inhibiting the production of INFs (**Figure 1.1**). On the other hand, HBV employs HBsAg to interact with PKM2, thereby promoting the expression of the downstream metabolic product lactate and subsequently facilitating HBV virus replication (**Figure 1.2**).

**Figure 1:**
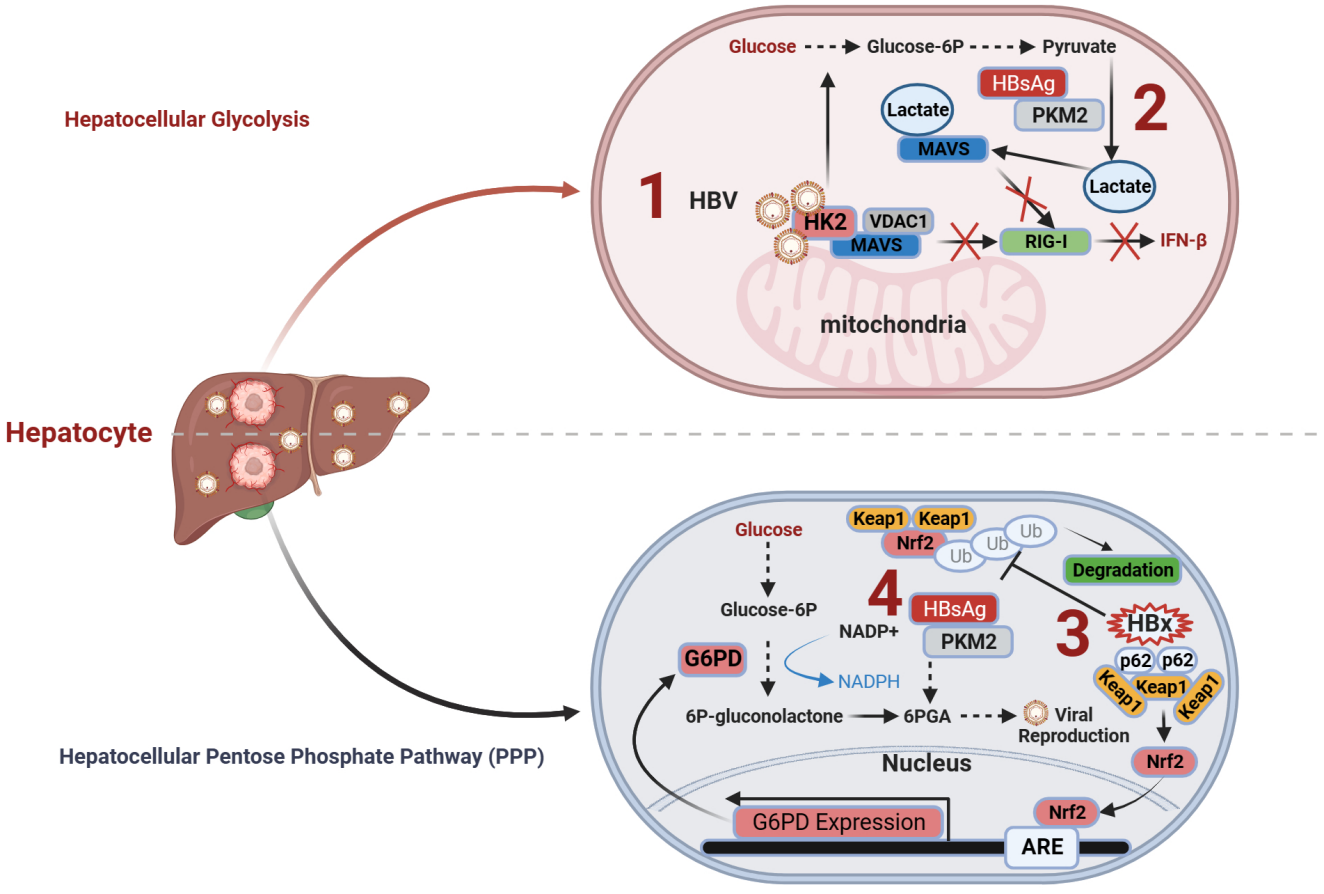
Immunometabolism and antiviral immunity of hepatocytes in CHB. **(1)** HBV induces the formation of the HK2-MAVS-VDAC1 trimeric complex and promotes the interaction between lactate and MAVS. This impedes the interaction between MAVS and RIG-I, suppressing downstream signal transduction and ultimately inhibiting the production of interferons; **(2)** HBV employs HBsAg to interact with PKM2, thereby promoting the expression of downstream metabolic product lactate and subsequently facilitating HBV virus replication; **(3)** The formation of HBx-p62-Keap1 aggregates sequesters Keap1 from Nrf2 and facilitates Nrf2 activation, forming the basis of HBV-induced Nrf2-mediated G6PD expression, promoting PPP and HBV viral replication; **(4)** HBV employs HBsAg to promote the PPP through the interaction of HBsAg with PKM2, thereby providing an ample supply of precursors for nucleotide and amino acid synthesis required for viral replication, facilitating sustained HBV replication. G6PD, glucose-6-phosphate dehydrogenase; HK2, hexokinase 2; Keap1, Kelch-like ECH-associated protein 1; MACS, mitochondrial antiviral signaling; Nrf2, NF-E2-related factor 2; PKM2, pyruvate kinase M2; RIG-I, Retinoic Acid-Inducible Gene I; VDAC1, voltage-dependent anion channel protein 1.

#### 2.1.2 Hepatocellular Pentose Phosphate Pathway (PPP)

The PPP is the primary route of glucose catabolic metabolism, encompassing the initial phases of glucose metabolism, along with two distinct metabolic stages. PPP directs glucose flux to its oxidative branch through the catalytic action of glucose-6-phosphate dehydrogenase (G6PD), which serves as the rate-limiting enzyme in this pathway. This process generates the reduced form of nicotinamide adenine dinucleotide phosphate (NADPH) (78).

HBV infection exerts multilevel effects on the PPP in liver cells, including alterations in glycolytic pathway activity (57) and the regulation of key enzyme activities (76). Seizing control of the host macromolecular metabolic supply is a common strategy employed by viruses for replication (41, 79). Different proteins encoded during HBV infection can affect the PPP within liver cells through distinct mechanisms. HBV enhances the expression of G6PD by activating Nrf2 (76), thereby promoting PPP. Mechanistically, the formation of HBx-p62-Keap1 aggregates sequesters Kelch-like ECH-associated protein 1 (Keap1) from NF-E2-related factor 2 (Nrf2) and facilitates Nrf2 activation, forming the basis for HBV-induced Nrf2-mediated G6PD expression. Nrf2 is a master transcription factor that regulates the expression of numerous antioxidant and cell-protective genes.

Under normal circumstances, Nrf2 associates with its inhibitor Keap1 and undergoes proteasomal degradation (80). Elevated Nrf2 activity amplifies the expression of enzymes within the PPP, including G6PD, thereby accelerating cancer cell proliferation (81). Intriguingly, investigations propose that the accumulation of p62, an autophagic adaptor protein, fosters sustained activation of Nrf2, thereby facilitating the progression of HCC (82, 83). In HBV infection, the HBV X protein (HBx) plays a pivotal role in forming a complex with p62 and Keap1, sequestering Keap1 within the aggregate, thereby preventing Keap1-mediated inhibition of Nrf2, promoting Nrf2 nuclear translocation, and enhancing the expression of the target gene G6PD, the rate-limiting enzyme of PPP (76). Additionally, the interaction between HBV and LHBS with PKM2 promotes aerobic glycolysis and activates PPP. A study found that inhibiting the formation of PKM2 dimers (increasing PKM2 activity) led to the reduced expression of 6-PGA, a component of PPP (57) (**Figure 1.4**). PPP upregulation provides precursors for nucleotide and amino acid biosynthesis, making it an ideal model to promote viral replication (57).

Altogether, HBV employs HBx to activate G6PD and LHBS-PKM2 to promote the PPP, thereby providing an ample supply of precursors for amino acid and nucleotide synthesis required for viral replication, facilitating sustained HBV replication (**Figure 1.3**). Additionally, HBV-induced intermediate metabolites of PPP may potentially alter the accessibility of the antiviral immune gene chromatin through epigenetic modification pathways. For further details, please refer to section 4.1.

### 2.2 Epigenetic modifications and antiviral immunity

Research on epigenetic modifications in CHB has focused primarily on cccDNA domains. Within the nucleus, the HBV DNA template adopts a minichromosomal structure that interacts with both histone and nonhistone proteins (84). Consequently, epigenetic regulatory mechanisms, including DNA methylation and histone modifications, can influence cccDNA activity, potentially offering new therapeutic concepts for treating chronic hepatitis B (CHB) (85, 86) (**Figure 2.1**). Conversely, HBV may induce alterations in the host genome at both the genetic and epigenetic levels (86). This dysregulation in gene expression may contribute to the development of CHB infection.

**Figure 2:**
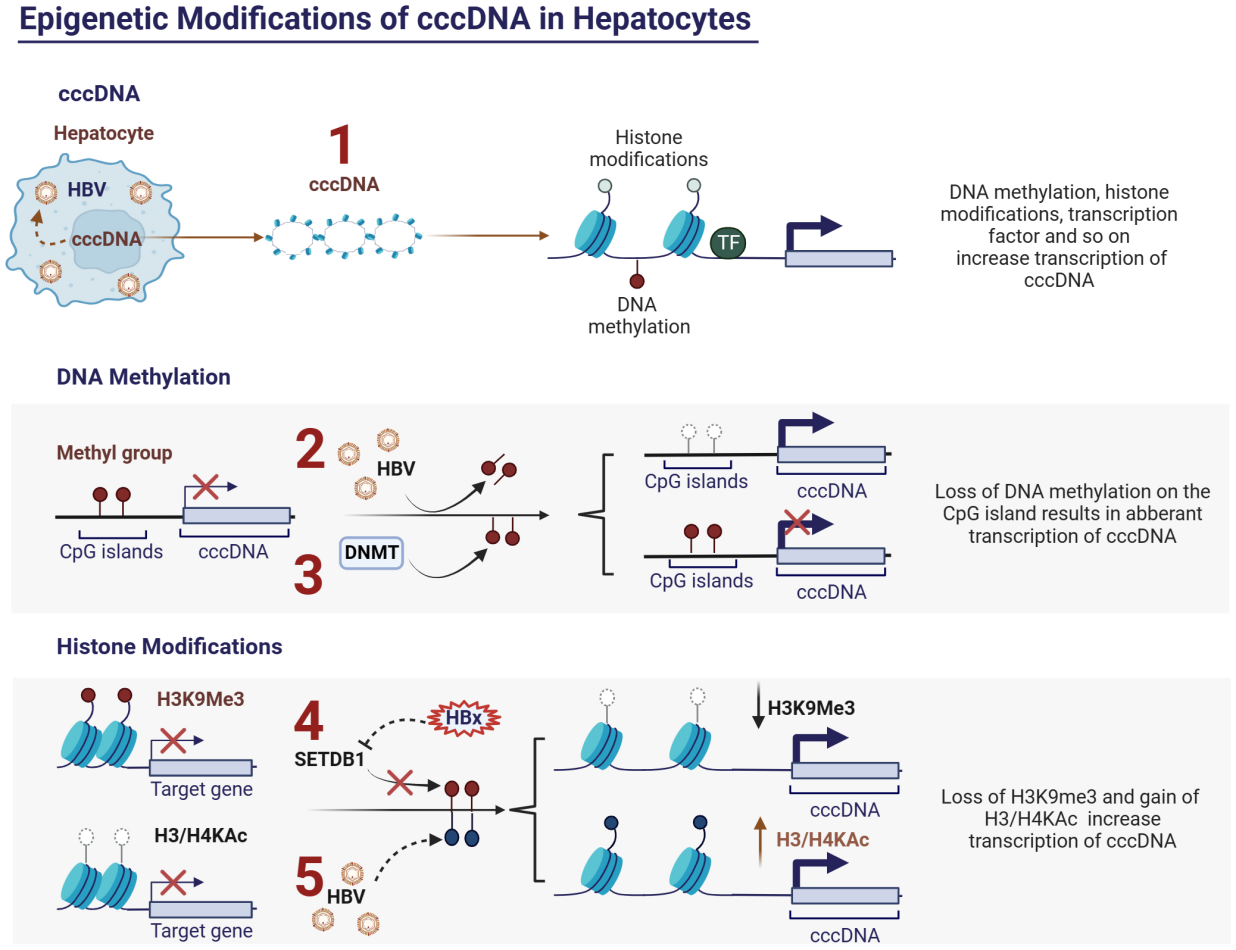
Epigenetic modifications of cccDNA in hepatocytes and HBV. **(1)** Activity of cccDNA regulated by epigenetic modifications, such as DNA methylation, histone modifications and so on; **(2)** HBV inhibits DNA methylation at CpG islands of cccDNA and thus promotes cccDNA transcription; **(3)** Upregulating the expression of DNA methyltransferases (DNMTs) can induce methylation of the viral genome, thereby inhibiting HBV replication; **(4)** HBx protein can relieve transcriptional silencing of cccDNA by slowing down the histone methylation (H3K9me3) upregulated by histone methyltransferase SETDB1, thereby promoting viral replication. **(5)** HBV can promote viral replication by modulating the acetylation levels of histones H3 and H4, which are associated with cccDNA. Ac, acetylation; cccDNA, covalently closed circular DNA; DNMTs, DNA methyltransferases; H3, histone 3; H4, histone 4.

#### 2.2.1 DNA methylation modification

DNA methylation occurs within specific CpG islands and is typically mediated by DNA methyltransferases (DNMTs), which is closely associated with transcriptional silencing (87). The life cycle of HBV has been extensively described in numerous articles (85). Briefly, HBV cccDNA is a single minichromosome with a chromatin-like structure containing four open reading frames (ORFs) encoding HBsAg, HBeAg, HBx protein, and viral polymerase (pol) (88–90). Depending on the genotype, cccDNA contains 2-3 CpG islands (91, 92) located at 1) the initiation site of the S gene; 2) the X gene and genome pre-RNA promoter; and 3) the pS1 and polymerase gene promoters (84). Among these, methylation at position 1 occurs infrequently and varies with genotype, whereas positions 2 and 3 are relatively conserved and more prone to methylation. Studies have demonstrated a clear negative correlation between the methylation level at position 2 and transcription and replication levels of cccDNA (93). Additionally, evidence suggests a significant elevation in the methylation levels at CpG island 2 in HBeAg-negative patients relative to infected and cirrhotic tissues. Moreover, the methylation levels of HBV DNA at CpG islands 2 and 3 in HCC tissues are higher than that in infected and cirrhotic tissues (91, 94, 95).

DNA methylation of the viral genome has been identified as a host defense mechanism (96). HBV infection can induce changes in host genome DNA methylation, leading to fluctuations in the methylation levels of specific genes, subsequently affecting their expression (97, 98) (**Figure 2.2**). This opens up the possibility of suppressing HBV replication by modulating the methylation levels of the host DNA. Upregulation of DNA methyltransferases (DNMTs) can induce methylation of the viral genome *in vitro*, thereby inhibiting HBV replication (99) (**Figure 2.3**). However, the host DNA methylation is also a major mechanism leading to the inactivation of tumor suppressor genes in HCC (100). Therefore, the precise regulation of the methylation levels of specific genes is a crucial topic for future research in the field of viral epigenetics.

#### 2.2.2 Histone modifications

Histone modifications, including methylation, acetylation, phosphorylation, and ubiquitination, regulate the accessibility of chromatin and consequently affect the expression levels of associated genes (101). Histone modifications play crucial regulatory roles in the course of CHB infection. HBV infection induces alterations in the histone modification patterns of specific genes, thus influencing the intensity and direction of the host immune response. Experimental evidence has demonstrated that HBV can promote viral replication by modulating the acetylation levels of histones H3 and H4, which are associated with cccDNA (102) (**Figure 2.5**). Additionally, there is evidence suggesting that the HBx protein can relieve transcriptional silencing of cccDNA by slowing down histone methylation (H3K9me3) upregulated by the histone methyltransferase SETDB1, thereby promoting viral replication (103) (**Figure 2.4**). Furthermore, specific histone modifications, such as acetylation and methylation, affect the transcriptional activity of immune cells, thereby accelerating immune escape (45). While epigenetic therapies show promise for the treatment of CHB, achieving precise control of epigenetic modifications of specific genes remains a crucial issue that urgently needs to be addressed.

## 3 Immunometabolism and epigenetic modification of immune cells during CHB

Viral infection disrupts the balance between sustained activation and inhibition of the host immune system, typically through the modulation of the metabolic state of immune cells (104). Similar adaptive changes in immune cell metabolism are also evident during CHB infection, subsequently influencing the effectiveness of host immune responses, involving monocytes/macrophages, kupffer cells, dendritic cells, natural killer cells (NK cells), T cells, and B cells (105–108).

### 3.1 Monocytes/macrophages

Monocytes/Macrophages, as the primary line of defense against infections, are almost ubiquitously present in various tissues under steady-state physiological conditions. Monocytes/Macrophages exhibit high plasticity and heterogeneity (109). For instance, hepatic macrophages are highly sensitive to the hepatic microenvironment and can rapidly adapt their phenotype (110). HBV infection can polarize macrophages toward an immunosuppressive M2 phenotype, inhibiting the secretion of inflammatory cytokines and stimulating the secretion of the anti-inflammatory cytokine interleukin-10 (IL-10) (111), thereby limiting innate immunity and promoting HBV immune evasion. Consistent with our previous studies, we observed that HBV affects the cytokine secretion functionality of monocytes/macrophages and attenuates their antiviral innate immune responses (112–114).

Metabolism governs the differentiation and function of macrophages (115), whereas epigenetic modifications can directly impact immune function by regulating the activity of metabolic enzymes, thereby influencing energy metabolism (116, 117). Recent studies have demonstrated that HBV-induced hyperacetylation of citrate synthase (CS) and the pyruvate dehydrogenase complex (PDHC) negatively regulates innate immune responses by impairing the TCA cycle in macrophages (105). Mechanistically, HBV activates the TLR2-NF-κB-PGC-1α axis in macrophages, leading to downregulation of the deacetylase SIRT3, thereby increasing acetylation of CS/PDHC and inhibiting their activity, ultimately disrupting the TCA cycle and promoting M2-like polarization of macrophages (105). Additionally, activation of signaling pathways is closely associated with macrophage polarization. AKT signaling activation promotes polarization toward the M2 phenotype, while NF-κB signaling activation suppresses M2 phenotype (118, 119). A recent study on chronic HBV infection suggested that HBV facilitates M2 macrophage polarization through SIRT1-mediated NICD deacetylation, resulting in reduced pro-inflammatory tumor necrosis factor α (TNF-α) and enhanced anti-inflammatory IL-10 expression (120). Mechanistically, HBsAg and HBeAg upregulate the expression of SIRT1 deacetylase, promoting NICD deacetylation, thereby enhancing Akt phosphorylation and inhibiting NF-kB nuclear translocation, ultimately driving M2 macrophage polarization (120).

In summary, HBsAg and HBeAg can, on one hand, upregulate the deacetylase SIRT1, facilitating NICD deacetylation, enhancing Akt phosphorylation, and inhibiting NF-kB nuclear translocation, thereby inducing the formation of immunosuppressive macrophages. On the other hand, HBV can also downregulate the expression of the deacetylase SIRT3 through the TLR2-NF-κB-PGC-1α axis, thereby increasing the acetylation of metabolic enzymes CS/PDHC and inhibiting their activity. This leads to impairment of the TCA cycle and promotion of the M2 phenotype in macrophages (**Figure 3.1**).

**Figure 3:**
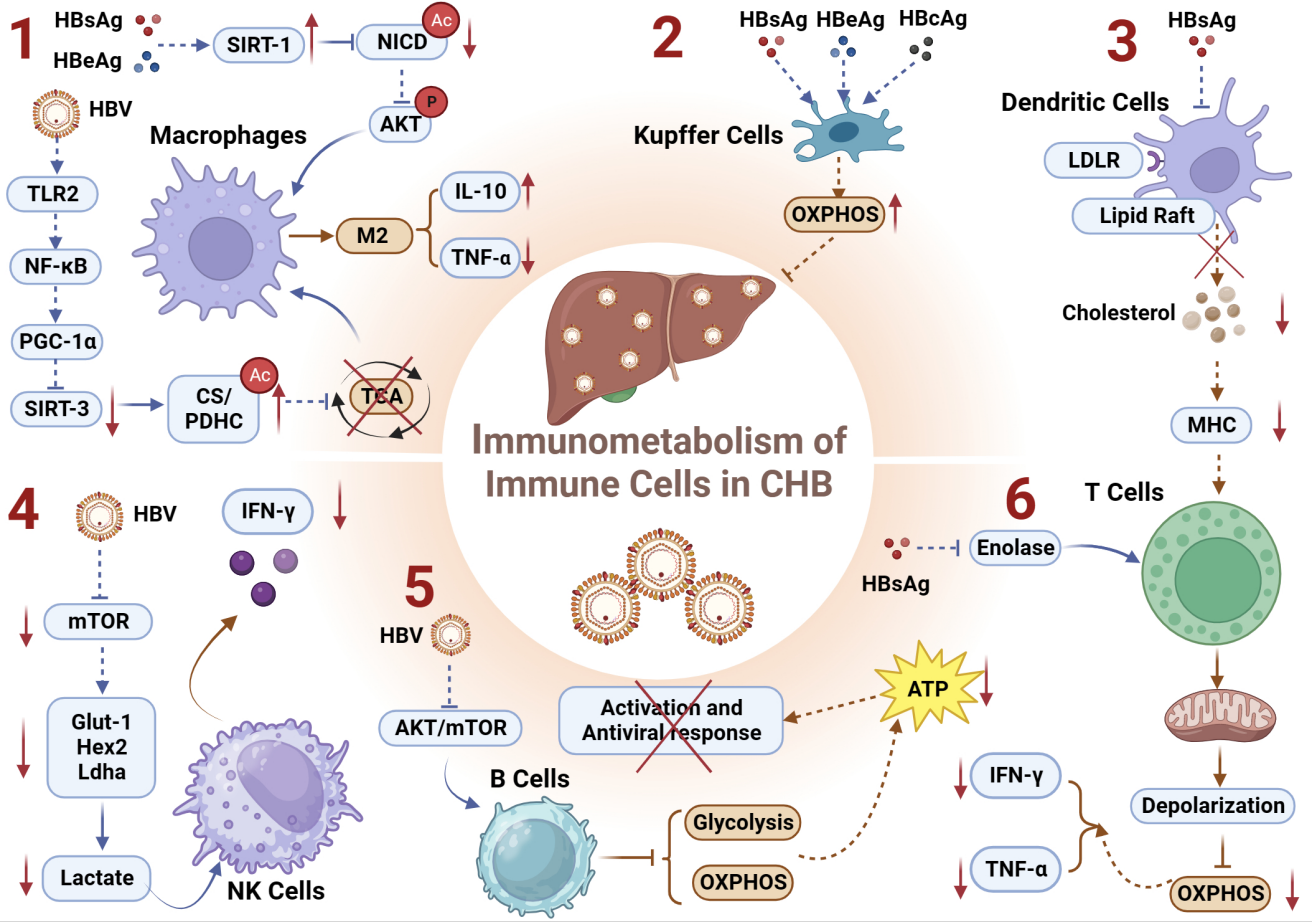
Immunometabolism of Immune Cells and Chronic HBV infection. **(1)** HBsAg and HBeAg upregulates the deacetylase SIRT1, facilitating NICD deacetylation, enhancing Akt phosphorylation, and inhibiting NF-kB nuclear translocation, thereby inducing the formation of immunosuppressive macrophages; HBV downregulates deacetylase SIRT3 through the TLR2-NF-κB-PGC-1α axis, thereby increasing the acetylation of metabolic enzymes CS/PDHC and inhibiting their activity. This leads to impairment of the TCA cycle and promotion of the M2 macrophages manifested by increased IL-10 and decreased TNF-α; **(2)** HBsAg, HBcAg, and HBeAg can induce immunometabolic reprogramming in KCs, upregulating OXPHOS and thereby inhibiting the host’s antiviral response; **(3)** HBsAg inhibits lipid raft formation and/or LDLR-mediated endocytosis on DCs, resulting in reduced levels of free cholesterol during CHB. This inhibition of antigen presentation capacity in DCs induces exhausted HBV-specific CD8^+^ T cells; **(4)** HBV regulates the Glut1-Hex2-Ldha-lactate axis-mediated metabolic reprogramming by inhibiting the metabolic regulatory factor mTOR pathway, thereby suppressing the expression of IFN-γ in NK cells; **(5)** HBV virus impairs the glycolytic and OXPHOS metabolic functions of B cells by inhibiting the AKT/mTOR pathway, and leads to a reduction in ATP production attenuating the activation and the antiviral immune response of B cells; **(6)** HBsAg can inhibit the key metabolic enzyme enolase, leading to depolarization and impaired OXPHOS function of exhausted T cell mitochondria, manifested as decreased secretion of IFN-γ and TNF-α. CS, citrate synthase; DCs, Dendritic Cells; GLUT-1, glucose transporter-1; IL-10, interleukin-10; KCs, Kupffer Cells; LDLR, low-density lipoprotein receptor; OXPHOS, oxidative phosphorylation; PDHC, pyruvate dehydrogenase complex; TNF-α, tumor necrosis factor α.

### 3.2 Kupffer cells

The liver is host to the largest population of tissue-resident macrophages, recognized as Kupffer cells (KCs). During CHB, the HBV employs diverse strategies to manipulate KCs, facilitating infection establishment. This includes inducing KCs to attenuate B cell antibody production (121), inhibiting CD8^+^ T cell cytotoxicity (122), and preferentially producing profibrogenic/anti-inflammatory cytokine TGF-β1 (123) to foster hepatic immune tolerance. A murine study investigating maternally transmitted HBV and its role in vertical virus transmission revealed that HBV can induce an M2-like anti-inflammatory polarization in KCs (124). Subsequent research within this study demonstrated that both M1- and M2-like KCs stimulated by HBV have the capacity to directly inhibit HBV replication in hepatic cells. Mechanistically, the study identified HBV-associated antigens, including HBsAg, HBcAg, and HBeAg, as capable of reprogramming KCs’ metabolism to enhance their OXPHOS activity (**Figure 3.2**). This reprogramming minimizes host antiviral responses (125). However, the precise mechanisms by which HBV orchestrates metabolic reprogramming in KCs and the impact of immunometabolic alterations on their epigenetic modifications remain elusive. Further investigations in this field hold the potential to unveil novel insights and contribute to a deeper understanding of the intricate interplay between HBV and KCs (125).

### 3.3 Dendritic cells

As proficient antigen-presenting cells (APCs) integral to the immune system, dendritic cells (DCs) play a pivotal role in bridging the innate and adaptive immune responses (126). The intricate regulation of immunometabolism is recognized as a key determinant influencing the functionality and viability of immune cells, with DCs relying on finely tuned immunometabolic processes to either initiate immune responses or foster immunological tolerance (127). Contrasting the characteristics observed in healthy donors (HD), DCs derived from individuals with CHB manifest impaired antigen presentation capabilities and diminished migratory proficiency (128). A recent investigation within the realm of DCs’ immunometabolism has revealed that HBsAg hampers lipid raft formation and/or low-density lipoprotein receptor (LDLR)-mediated endocytosis on DCs, culminating in diminished levels of free cholesterol during the course of CHB. This impediment in the antigen presentation capacity of DCs precipitates the exhaustion of HBV-specific CD8^+^ T cells, thereby fostering the protracted presence of HBV (129) (**Figure 3.3**). The findings underscore the intricate interplay between immunometabolism and viral persistence, shedding light on a mechanistic link between HBsAg-mediated alterations in DC function and the sustained evasion of host immune surveillance by HBV.

Cholesterol stands as an essential lipid molecule intricately involved in pivotal biological processes, encompassing lipid rafts and major histocompatibility complex (MHC) molecules (130). Strategies aimed at increasing cholesterol accumulation on DCs can enhance antigen presentation, thus improving the efficacy of therapeutic vaccines and rescuing the exhausted HBV-specific CD8^+^ T cells (129).

### 3.4 NK cells

Natural killer (NK) cells, as another integral component of the innate immune system (131), play a crucial role in defending against pathogenic infections (132). Our previous research has revealed that HBV utilizes monocytes to modulate NK cells, thereby affecting their regulatory functions and antiviral immunity (112, 113). NK cells secrete TNF-α and IFN-γ, which may contribute to the cytotoxicity involved in HBV clearance (133). In patients with acute HBV infection, NK cells are activated during the acute phase before the adaptive immune response kicks in (134–138). However, during the chronic phase of the disease, NK cells exhibit impaired functionality, which may facilitate viral persistence. Indeed, NK cells from CHB patients show reduced capacity to produce cytokines such as TNF-α and IFN-γ, despite maintaining or even augmenting their cytotoxic capabilities (139–142), a phenomenon referred to as functional dichotomy (139). Additionally, NK cells play crucial roles in shaping and influencing adaptive immune responses by exerting immunomodulatory effects (143).

However, the immune capacity of NK cells at different stages of infection is closely linked to their metabolic phenotypes (144–146). Functional deficiency of peripheral NK cells in patients with CHB is associated with defects in a key metabolic regulatory factor, the mTOR pathway (106). This is primarily manifested by a significant inhibition of cytokine secretion, particularly IFN-γ, while the cytotoxic function remains unaffected in the same environment. The mTOR-mediated metabolic reprogramming has been reported to be closely associated with the function of NK cells (147, 148). Inhibiting mTOR signaling can reduce the rate of glucose uptake and glycolysis in NK cells by inhibiting the key rate-limiting enzymes involved in glycolysis, such as Glut1, Hex2, and Ldha, thereby suppressing lactate production. Furthermore, this pathway is closely related to the expression of IFN-γ in NK cells (149).

Recent studies indicate that epigenetic reprogramming may play a central role in the persistent phenotypic and functional changes in the effector and memory NK cell populations (150). During human cytomegalovirus (HCMV) infection or reactivation, the expanded and host-protective regulated by memory-like NK cell subset demonstrates features of epigenetic reprogramming at genes involved in effector functions and intracellular signaling (151–153). For instance, the enhanced IFN-γ response in these memory-like cells is associated with decreased DNA methylation at the IFNG locus, while the reduced expression of signaling molecules FcϵRIγ and EAT-2 correlates with increased DNA methylation at gene loci (152, 153). Recent ATAC-seq and ChIP-seq studies reveal extensive and highly dynamic epigenetic reprogramming in Ly49H NK cells during the progression of effector, contraction, and memory phases in response to murine cytomegalovirus (MCMV) infection (154). Changes in NK cell chromatin accessibility, particularly at promoter regions, have been shown to directly correlate with transcript abundance changes at early time points after MCMV attack (154), notably for genes related to type I and II interferon signaling, such as Ifng and various ISGs (154). Although there is currently no specific literature on the epigenetic modifications of NK cells under CHB infection, this presents a new avenue for investigating the specific mechanisms underlying NK cell functional defects during CHB infection.

Taken together, HBV regulates the Glut1-Hex2-Ldha-lactate axis-mediated metabolic reprogramming by inhibiting the metabolic regulatory factor mTOR pathway, thereby suppressing the expression of IFN-γ in NK cells (**Figure 3.4**). Exploring whether HBV regulates epigenetic reprogramming of interferon genes (e.g., methylation, lactylation modifications) by inhibiting the metabolic regulator mTOR pathway and thereby influencing the antiviral function of NK cells would be an intriguing research direction.

### 3.5 T cells

T cell exhaustion is a prominent feature of CHB infection (155–158). Energy metabolism in T-cells is closely associated with the function of effector T cells. When T cells experience nutrient deficiency or the inhibition of key metabolic enzymes, their activation and proliferation are suppressed (159–161). Extensive research has highlighted the significant role of glycolytic enzymes in T cell function and activation, such as the crucial glycolytic metabolite phosphoenolpyruvate (PEP) (162) and others (163–165). During HBV infection, despite significantly elevated glucose uptake in severely exhausted virus-specific CD8^+^ T cells, there is impaired oxidative phosphorylation, subsequently affecting their ability to secrete IFN-γ and TNF-α, which correlates with HBsAg levels (107).

In terms of mechanism, HBsAg may inhibit the expression of enolase in exhausted T cells, thereby restricting glycolytic flux. This effect can be enhanced by supplementing downstream product pyruvate (PEP) (107). Additionally,during HBV infection, functional and non-exhausted T cells can utilize oxidative phosphorylation (OXPHOS) to meet their energy demands. In contrast, exhausted T cells exhibit dysfunctional depolarized mitochondria despite increased expression of glucose transporter-1 (GLUT-1), indicating impaired OXPHOS function, which subsequently affects the antiviral immune function of T cells (166). Consequently, the compromised functionality of HBV-specific CD8^+^ T cells does not stem from a deficiency in glucose uptake but rather from their diminished ability to engage OXPHOS to meet their energy demands. This deficiency hampers the capacity of T cells for metabolic reprogramming, transitioning from glycolysis to OXPHOS, which is essential for fulfilling the bioenergetic requisites crucial for the establishment of protective T cell memory (167). Recent investigations have further validated this underlying mechanism, with studies indicating that addressing mitochondrial dysfunction can effectively restore the antiviral activity of exhausted HBV-specific CD8^+^ T cells in CHB (168).

A recent authoritative review on the metabolic and epigenetic regulation of T cell exhaustion proposed that changes in mitochondrial adaptability and dynamics may drive epigenetic alterations that enhance T cell exhaustion, further impeding T cell immune function by influencing chromatin accessibility (169). This review highlights acetyl-CoA as a plausible candidate with potential supporting evidence. Under aerobic conditions, pyruvate generated from glycolysis enters the tricarboxylic acid (TCA) cycle in the mitochondria to produce acetyl-CoA [31], and the levels of acetyl-CoA are highly dependent on glucose availability and mitochondrial respiratory function (170). Acetyl-CoA forms the basis for the functions of histone acetyltransferases (HATs) (53). Evidence has long established that the availability of acetyl-CoA regulates many protein acetylation modifications (52, 171, 172). A mouse study demonstrated that reducing the levels of acetyl-CoA led to decreased acetylation of lysine 9 residues on histone H3 at the IFNG gene locus in CD4^+^ T cells, thereby suppressing IFNG expression (59). Additionally, promoting histone acetylation and chromatin accessibility can restore IFN-γ secretion in CD8^+^ T cells under glucose-restricted conditions (173).

Together, HBsAg can inhibit the key metabolic enzyme enolase, leading to the depolarization of exhausted T-cell mitochondria, manifested as impaired OXPHOS function (**Figure 3.6**). This specific alteration in metabolic reprogramming may lower acetyl-CoA levels, subsequently inhibiting the acetylation levels of target genes (IFN-γ and TNF-α), hindered the antiviral immune function of T cells.

### 3.6 B cells

Functioning as essential components of the humoral immune system, B cells play a pivotal role in generating antibodies against HBV (174). Notably, recent studies have focused on investigating deficiencies in both intrahepatic and circulating antiviral B cell responses in individuals with hepatitis B (175–177). Persistent viral infections can compromise the various antiviral functions of B cells, including antigen presentation, humoral immunity, and cytokine production (178–180). The activation status of B cells is closely related to their metabolic pathways. Unlike T cells, activated B cells lead to a wide-ranging increase in metabolism, including enhanced oxygen consumption and extracellular acidification rates (181).

During CHB infection, the virus inhibits the metabolic capacities of B cells, including OXPHOS and glycolysis. This, in turn, affects the energy supply and effector secretion of B cells, leading to a decrease in their proliferation and differentiation abilities, thereby facilitating the sustained presence of HBV (108). Mechanistically, HBV suppresses the AKT/mTOR pathway in B cells, reducing their glucose uptake and causing mitochondrial dysfunction, specifically by impairing OXPHOS function (108). Because OXPHOS provides 85% of the total ATP in activated PBMCs (182), HBV virus impairs the production of ATP in B cells by compromising both glycolysis and OXPHOS functions, thereby attenuating the activation of B cells.

Additionally, epidemiological studies have shown an increased risk of Non-Hodgkin’s lymphoma (NHL) in individuals with CHB infection, indicating a significant correlation (183, 184). Furthermore, recent clinical cohort studies on HBV and B cells have identified a higher occurrence of mutations in the key epigenetic regulator, ARID1A, in HBV cases (185, 186). Notably, HBV-related HCC also exhibits a high frequency of ARID1A mutations (187). ARID1A encodes a subunit of the Switch/Sucrose-Nonfermentable (SWI/SNF) chromatin remodeling complex, which primarily regulates chromatin accessibility to allow for the activation of target gene transcription. Importantly, its function in chromatin remodeling is ATP-dependent (188). Subunits of the SWI/SNF complex are crucial for the expression of B lineage-specific genes and the development of B cells (189).

Collectively, HBV impairs the glycolytic and OXPHOS metabolic functions of B cells by inhibiting the AKT/mTOR pathway (**Figure 3.5**). This inhibition leads to a reduction in ATP production, and the expression of the ATP-dependent epigenetic modification complex (SWI/SNF) responsible for promoting chromatin accessibility, ultimately affecting the antiviral immune response of B cells.

## 4 Discussion

This review aimed to examine how HBV affects the immunometabolism and epigenetic reprogramming of crucial cells, such as hepatocytes and immune cells, in the hepatic immune microenvironment. By altering the immunometabolism and epigenetic modifications in these cells, HBV impairs antiviral immune responses and facilitates the chronic persistence of HBV infection. However, some intriguing aspects of this process warrant further attention as they may offer novel insights into the pathogenic mechanisms of HBV.

### 4.1 Potential connections between immunometabolism, epigenetic modifications, and antiviral immunity in HBV-infected hepatocytes?

In HBV-infected hepatocytes, the virus can modulate the epigenetic modifications of downstream target genes (such as IFN) by regulating immunometabolism, thereby exerting an impact on antiviral immunity. HBV influences the antiviral immune response and viral replication in hepatocytes by intervening in the glycolytic and PPP metabolic pathway (**Figure 1**). Specifically, the primary outcomes of the impact of HBV on hepatocyte glycolysis are an increase in the metabolic product lactate and suppression of IFN production (58). Coincidentally, lactate derived from glycolysis acts as a threshold inhibitor of IFN production when RLR is activated, providing strong evidence of a negative regulatory relationship between lactate and IFN (190). This underscores the importance of lactate as a crucial metabolic product in innate immune responses in hepatocytes during CHB infection. Notably, a new epigenetic modification, lactylation, was discovered four years ago, elevating our understanding of lactate to unprecedented levels (51). Lactate-driven lactylation modifications play a vital role in suppressing inflammation and immune processes, making them a popular topic in recent research (191, 192). Therefore, exploring whether lactate, through its induced lactylation modifications, reduces the accessibility of IFN gene chromatin, thereby inhibiting the antiviral immune response in hepatocytes, would be an intriguing research direction. Lactate-induced lactylation may serve as a bridge between immunometabolism and antiviral effects.

Concomitantly, HBV promotes the hepatocellular PPP metabolism and sustains viral replication (57, 76). One of the main functions of the PPP is the generation of NADPH (193), which is an indispensable reducing agent in synthetic metabolic processes. The upregulation of HBx in hepatocytes increases the production of intracellular ATP and NADPH (194). Recent research revealed that NADPH can interact with histone deacetylase 3 (HDAC3), inhibiting its activity by disrupting its binding with co-factors (195). Simultaneously, in studies on hepatocytes, overexpression of the deacetylase HDAC3 can inhibit HBV replication (196). Recent studies have found a direct correlation between deacetylase and the production of type I interferons in innate immunity. In U3OS cells infected with Sendai virus (SeV), acetyl groups are present at specific DNA-binding domain (DBD) sites of IRF3/IRF7, thereby eliminating the liquid-liquid phase separation (LLPS) and IFN-I induction of IRF3/IRF7 (197). In other words, inhibiting deacetylases can block IFN signal transduction, thereby promoting viral replication. This further supports the conclusion that HBV protein expression interferes with the transmission of type I IFN signaling in infected hepatocytes (198). In summary, HBV increases the accumulation of NADPH, a product of PPP metabolism, in hepatocytes. NADPH may potentially interact with HDAC3, inhibiting deacetylase activity, which promotes the acetylation of the DNA-binding domain (DBD) of IRF3/IRF7. This, in turn, suppresses IFN expression and facilitates viral replication.

### 4.2 Could there be a connection between the immunometabolism, epigenetic modifications, and immune function of immune cells during CHB?

From a metabolism-epigenomics perspective, metabolites can serve as substrates or cofactors for epigenetic modifications, thereby altering the structure and function of chromatin and genomic regions associated with diseases (55). Epigenetic modifications can also directly impact energy metabolism and thus influence immune function by either regulating the transcription levels of metabolic enzymes or affecting the activity of metabolic transcription factors (117, 199). This intricate interplay creates a complex network between immunometabolism and epigenetics in CHB infection. In our recent study, we found that HBV can induce immunometabolism and epigenetic reprogramming in monocytes/macrophages, subsequently influencing their functionality and fate (to be published). Therefore, in-depth research into immunometabolism and epigenetic reprogramming will provide new perspectives for understanding the mechanistic origins of CHB.

Furthermore, as described above, exploring the relationships between immunometabolism and epigenetic modifications in various immune cells will be a captivating and highly meaningful research direction. Firstly, it remains an intriguing question whether HBV modulates the epigenetic reprogramming of interferon genes, such as methylation and lactylation, by inhibiting the metabolic regulatory factor mTOR pathway, consequently impacting the antiviral function of NK cells. Secondly, despite HBV inducing specific T cell exhaustion, leading to mitochondrial dysfunction and affecting the secretion of IFN-γ and TNF-α (107), the intricate mechanisms underlying the metabolic reprogramming and altered antiviral activity in T cells remain elusive. The potential reduction of acetyl-CoA levels in T cells through HBV-induced OXPHOS metabolic reprogramming may inhibit the acetylation levels of target genes, such as IFN-γ and TNF-α, representing a profound mechanism underlying the immune deficiency in exhausted T cells. Lastly, HBV, by suppressing the glycolysis of B cells, not only impairs the activation of B cells but also inhibits ATP levels within B cells and the expression of the ATP-dependent epigenetic modification complex SWI/SNF, both of which may synergistically further compromise the antiviral immune response of B cells.

### 4.3 The dialogue at the immunometabolic and epigenetic levels between hepatocytes and immune cells within the hepatic immune microenvironment

HBV affects metabolic reprogramming of hepatocytes through various pathways, leading to increased lactate production (57, 58). Lactate is a metabolic intermediary that shapes the fate and function of immune cells (200). Lactate plays a key role in oxidation, gluconeogenesis, and cellular signal transduction (201–203). Endogenous lactate is primarily produced through aerobic or anaerobic glycolysis, whereas exogenous lactate primarily enters the cells via monocarboxylate transporters (MCTs) (204) and G protein-coupled receptor 81 (GPR81) (GPR81) (205). MCT-1 mediates the effects of lactate on macrophages and CD8^+^ T cells (50, 206), potentially providing lactate as a messenger from hepatocytes to regulate immune cells. A decade ago, research indicated that exogenous lactate could induce the polarization of macrophages toward the M2 phenotype, manifested by increased expression of VEGF and ARG1 (50). Similarly, recent findings suggested that lactylation modifications specifically induced by lactate promote the expression of VEGF and ARG1 in macrophages (51). Furthermore, in the field of lactate research, predominantly focused on tumors, physiologically relevant concentrations of lactate can inhibit the activation of CD8^+^ T and NK cells, resulting in reduced production of IFN-γ (207). Tregs can absorb and metabolize lactate, thereby sustaining their suppressive function in environments characterized by elevated lactate levels, as observed in contexts such as the tumor microenvironment (208). Whether lactate-mediated inhibition of IFN-γ secretion in NK and T cells is facilitated through lactylation modifications presents an intriguing avenue for exploration.

## 5 Concluding remarks and future perspectives

In this review, we comprehensively explored the interplay and pivotal roles of immunometabolism and epigenetic modifications within the hepatic immune microenvironment across various cell types in CHB infection research. Immunometabolism is considered the link between cellular metabolic status and innate immune function, which is crucial for responding to HBV infection and contributing to the inability of the immune system to resolve CHB. HBV replication forms a stable viral cccDNA minichromosome within the nucleus of liver cells, and epigenetic modifications in cccDNA facilitate persistent infection. Furthermore, the accumulation or depletion of metabolic products not only directly affects the functionality and dynamic balance of immune cells, but also serves as a substrate that regulates epigenetic modifications. This, in turn, influences the expression of antiviral immune genes, thereby promoting sustained HBV infection. By delving into the regulation of host immune metabolic pathways and the impact of epigenetic modifications in response to HBV infection, we not only gain a better understanding of the mechanisms underlying the development of CHB infection but also provide robust theoretical support for the development of novel therapeutic strategies.

Based on research findings on immunometabolism and epigenetic regulation, future developments may lead to personalized therapeutic approaches (209). Further investigations into the functions and regulatory mechanisms of key metabolic enzymes in immune metabolic pathways will contribute to the discovery of novel therapeutic targets for CHB infection. Additionally, a deeper understanding of the dynamic changes in epigenetic modifications during HBV infection and their association with disease progression may lead to the development of therapeutic strategies targeting specific gene-modifying enzymes. Precise analysis of patient metabolic and epigenetic characteristics can facilitate the design of targeted treatment plans, thereby enhancing treatment efficacy and alleviating patient suffering.

The field of immunometabolism and epigenetic modifications in CHB infection has opened new avenues for understanding and treating this disease. We hope that more rigorous and comprehensive research in this area will yield novel insights and strategies to combat the serious health challenges posed by CHB infections.

## Author contributions

ZW: Formal analysis, Resources, Writing – original draft, Writing – review & editing. NL: Conceptualization, Methodology, Supervision, Writing – review & editing. YY: Formal analysis, Resources, Supervision, Writing – review & editing. ZT: Conceptualization, Investigation, Methodology, Project administration, Resources, Writing – review & editing.
